# Post-traumatic Stress Disorder in Sexually Abused Children: Secure Attachment as a Protective Factor

**DOI:** 10.3389/fpsyg.2021.646680

**Published:** 2021-07-27

**Authors:** Karin Ensink, Peter Fonagy, Lina Normandin, Abby Rozenberg, Christina Marquez, Natacha Godbout, Jessica L. Borelli

**Affiliations:** ^1^Pavillon Félix-Antoine-Savard, Université Laval, Quebec, QC, Canada; ^2^University College London, London, United Kingdom; ^3^Department of Psychological Science, Social and Behavioral Sciences Gateway, University of California, Irvine, Irvine, CA, United States

**Keywords:** attachment, childhood sexual abuse, post-traumatic stress disorder, middle childhood, secure attachment

## Abstract

The aim of the present study was to examine the hypothesis that attachment and childhood sexual abuse (CSA) interacted such that school aged CSA survivors with insecure attachment to parents would be at an elevated risk of developing post-traumatic stress disorder (PTSD) and trauma symptoms. Participants (*n* = 111, ages 7–12) comprised two groups, child CSA survivors (*n* = 43) and a matched comparison group of children (*n* = 68) recruited from the community. Children completed the Child Attachment Interview (CAI) as well as the Trauma Symptom Checklist for Children (TSCC). There was a significant interaction between sexual abuse history and attachment security, such that sexually abused children with insecure attachment representations had significantly more PTSD and trauma symptoms than sexually abused children with secure attachment to parents. The findings show that using a dual lens of attachment and CSA can facilitate the identification of children most at risk and has important implications for understanding risk and resilience processes.

## Introduction

Childhood Sexual Abuse (CSA) is a major public health problem and human rights issue such that 20% of women and 5–10% of men report being sexually abused as children (Norman et al., 2012). CSA is associated with an increased risk of a wide range of trauma symptoms including post-traumatic stress disorder (PTSD; Briere, 2004; Briere et al., 2008). There is growing interest in the role of attachment in the pathways to recovery and dysfunction following trauma (Mikulincer et al., 2015) and how attachment style may play a role in precipitating, perpetuating, and protecting against Post Traumatic Stress (Bonanno, 2004; de Zulueta, 2006). As Mikulincer et al. (2006, p. 8) propose: “the mental health implications of attachment-system functioning are highly pertinent to understanding a person’s psychological reactions to traumatic events.” Trauma is theorized to activate the attachment system and the need for protection, so that there is a reciprocal relationship between attachment and trauma (Lieberman and Amaya-Jackson, 2005; Mikulincer et al., 2006). Lieberman and Amaya-Jackson (2005) propose using a “dual lens” focusing on both trauma and attachment to identify protective and aggravating processes involving these two major influences on children’s development. In order to restore a sense of security and reduce the impact of trauma, children as well as adults may seek out attachment figures or activate representations of internalized attachment figures. Secure attachment may have restorative effects after trauma and has been referred to as a protective shield (Lieberman and Amaya-Jackson, 2005). Consistent with this, Pynoos et al. (2009) argue that some PTSD manifestations may derive from the experience of threat and danger, whereas others derive from the lack of finding safety, as these systems are associated with distinct neurobiological systems.

Attachment has been shown to influence how traumatic reactions are experienced and expressed in adults (Mikulincer et al., 2015) and to impact the development of PTSD (Woodhouse et al., 2015; Barazzone et al., 2019). Attachment is associated with the number of PTSD symptoms, negative affectivity, somatization, emotional coping, attributions, and social support (O’Connor and Elklit, 2008). Furthermore, insecure attachment is associated with more severe PTSD, while secure attachment is associated with increased resilience, and may to some extent protect individuals from the negative effects of trauma (Bonanno, 2004). In adult CSA survivors, attachment security is a protective factor against the development of trauma symptoms (Aspelmeier et al., 2007) and attachment was found to mediate the relationship between CSA and trauma symptoms (Roche et al., 1999). Similarly, in adolescents, secure attachment moderates the relation between CSA and trauma symptoms (Jardin et al., 2017).

No previous studies have examined the associations between child attachment and PTSD and trauma symptoms in school-aged child CSA survivors. Studies with child CSA survivors referred for treatment show that 80% manifest some PTSD symptoms, but few meet the full criteria of PTSD (McLeer et al., 1992). Depending on the respondents (parent, child, or clinician) and how CSA criteria was defined studies indicate that 20–35% (Gospodarevskaya and Segal, 2012) or 8–19% (Maikovich et al., 2009) of child CSA survivors in the community meet PTSD criteria. Insecure attachment to parents was found to be a risk factor for child-reported depressive symptoms in school aged child CSA survivors (Ensink et al., 2019). Similar research is needed to examine interactions between CSA and attachment to understand pathways to developing PTSD and trauma symptoms.

## Attachment

Attachment security, or the subjective sense that others will be responsive to one’s expression of needs for comfort and support, is thought to develop as a result of having received sensitive care from attachment figures (Bowlby, 1980). Based on early interactions between infant and caregiver, children develop cognitive-affective schema, referred to as Internal Working Models (IWM) that contains important expectations regarding the experience and expression of emotion and the responsiveness and reactions of others (Bowlby, 1980): When children’s expressions of emotional need have been met consistently with empathy and assistance in regulating emotion, children internalize the message that painful emotional experiences can be experienced, expressed, responded to and regulated. This facilitates optimal self-regulation of emotion later in development (Cassidy, 1994). In contrast, when children’s needs have been rejected or ignored or when caregivers have responded inconsistently or with alarm to children’s needs, children resort to defensive emotion regulation strategies, such as deactivation or hyperactivation. This may be adaptive in the short-term but can result in negative outcomes over the long-term (Cassidy, 1994). Decades of research substantiate this theorizing by documenting links between attachment security and emotion regulation in adults (e.g., Mikulincer et al., 2003; Mikulincer and Shaver, 2008).

Middle childhood remains an understudied developmental phase with regard to attachment and its links with emotion regulation (Bosmans and Kerns, 2015), but emerging evidence suggests that school-aged children with secure attachment have better emotion regulation than their insecure counterparts (Kerns et al., 2007; Borelli et al., 2010). The association between attachment and child emotion regulation is thought to depend on early parent-child interactions involving physical/embodied regulation by the parent (Shai and Belsky, 2011, 2017). These interactions serve to calibrate the infant’s developing stress regulation system so that over time, emotional and physiological self-regulation is established (Fonagy et al., 2002), with the presence of the parent needed and sought only in contexts of threat or higher levels of distress. In addition, child expectancies of the parent’s availability and responsiveness to distress are reflected at a representational level in IWM’s of self and other. By middle childhood, processes associated with secure attachment (Campos et al., 1994) have facilitated the establishment of emotional regulation capacities through their early physiological impact on the development of the stress regulation system (Gross, 1998). At the same time, secure attachment relationships are associated with the continued support of emotional regulation through the actual availability of attachment figures and the support and protection they may provide in times of distress (Kerns, 2008; Gross, 2013). In addition, secure attachment relationships promote regulation at a representational level through expectancies and the imagined responsiveness and trustworthiness of attachment figures and others in times of need (Fonagy et al., 2007). Furthermore, attachment relationships facilitate the development of social-cognitive capacities such as mentalizing about self and others that support self-regulation and interpersonal functioning by making reactions to behavior predictable and understandable (Ensink et al., 2015, unpublished).

Traumatized children with insecure attachment may be more vulnerable to developing PTSD, because of difficulties accessing emotionally supportive interpersonal relationships and use social support to buffer the impact of trauma (Lynch and Cicchetti, 1998). In line with this the link between attachment anxiety and PTSD symptoms has been found to be mediated by low perceived levels of social support (Besser and Neria, 2012). Furthermore, research with adults show that insecure attachment may also contribute to more severe trauma symptoms and psychopathology because it is associated with non-optimal emotion regulation strategies (Midolo et al., 2020). For example, avoidant strategies where there is a suppression of emotion and over reliance on self may result in distress being unresolved (Mikulincer et al., 2006).

To address the current gaps in the literature, the aim of the present study was to examine whether attachment security to caregivers acted as a moderator of trauma symptoms among child CSA survivors. Based on previous findings with adolescents (Jardin et al., 2017), we anticipated that children with both CSA and insecure attachment would have the highest levels of psychopathology as compared to children with CSA and secure attachment.

## Materials and Methods

### Participants

The study protocol was approved by the university ethics committee prior to the inception of the study. All participants were recruited as part of a larger longitudinal study examining psychosocial difficulties in the context of CSA. Sexually abused children (*n* = 43) and their mothers were referred to the university clinic by doctors, social services, or mental health practitioners working at community health services and hospitals in the city and surrounding regions. The community comparison group (*n* = 68) was recruited through advertisements at Community Health Services and schools through pamphlets soliciting participation (as part of a comparison group) in a study on the impact of CSA. The comparison group was selected to broadly match the socio-demographic, age (within 6 months), and gender characteristics of the abused group. The demographic features of the resulting sample are described in Table 1. More than half (61.3%) of the participants were female and their mean age was 9.53 years (*SD* = 1.45 years; ranging from 7 to 12 years). Reflecting the socio-demographics of the region, 98% of the participants were Caucasian. The assessments took place at the university child and adolescent consultation service. To compensate participants for their time and cost, parents were offered a modest stipend to cover their transport costs and the children were invited to select a toy or small gift.

**Table 1:** Descriptive statistics for key study variables.

	Total		Male		Female	
	*M*	*SD*	*M*	*SD*	*M*	*SD*
Age	114.39	17.374	112.9	15.573	115.31	18.451
% Male	40%	----	----	----	----	----
Mother educational attainment	15.22	3.67	15.45	3.664	15.07	3.695
Household income	4.59	2.26	4.76	2.526	4.48	2.092
Children’s stress exposure	1.47	1.47	1.40	1.41	1.5118	1.50826
% CSA-exposed	39%	----	35%	----	41%	----
% Insecure attachment	50%	----	51%	----	49%	----
Posttraumatic symptoms	46.44	8.269	45.02	7.866	47.32	8.449
Anxiety symptoms	48.66	9.783	46.73	8.176	49.86	10.542
Depression symptoms	46.94	10.559	45.88	9.76	47.61	11.047
Anger symptoms	43.84	8.541	44.17	10.195	43.64	7.408
Overt dissociation symptoms	48.4	8.409	47.02	8.119	49.26	8.533
Sexual concerns symptoms	51.95	14.631	49.98	8.615	53.18	17.304

In the child CSA survivor group, 23% had experienced vaginal or anal penetration with a penis or object and 5% had experienced violent sexual abuse. In terms of frequency of the abuse, 46% experienced CSA on two or three occasions, and 36% experienced CSA on four or more occasions and 18% experienced CSA on one occasion. Perpetrators were family members in approximately 50% of cases and of these children who had experienced intra-familial abuse, the majority (60%) had been abused by their fathers, 22% by siblings, and 18% by stepparents. Of the children who had experienced extra-familial CSA, the majority had been abused by an acquaintance (67%) or by a member of the extended family (33%). In terms of sexual abuse disclosure, in 55% of cases children denounced the CSA; in the remainder of cases, parents (mother: 19%, father: 3%) or other family members (23%) denounced the abuse.

Upon arriving at the university clinic, parents provided informed consent and children provided informed assent before data was collected for this study; the consent/assent process involved informing participants that they could withdraw from study participation at any time or refuse to participate in any part of the study. After completing the consent/assent process, children completed the CAI (Shmueli-Goetz et al., 2004, unpublished) with a trained clinical research assistant. Clinical research assistants then administered the Trauma Symptoms Checklist (Briere, 1996), an interview assessing children’s trauma-related psychopathology.

### Measures

#### Children’s Abuse History

Information regarding CSA was based on medical and social work reports and information from police inquiries, including statements of admission by the abuser. Parents of comparison group children were interviewed about the child’s developmental history and traumatic life events to ensure children in the comparison group did not have CSA histories. We classified children into three groupings (no CSA history, extrafamilial CSA history, and intrafamilial CSA history), wherein intrafamilial abuse referred to abuse by members of the immediate family such as a father, father-figure (including the mother’s partner), a sibling, or a grandparent. This designation of extrafamilial vs. intrafamilial CSA was only employed in a set of preliminary analyses in which we examined whether the type of abuse was associated with attachment and trauma symptom outcomes.

#### Children’s Attachment Security

The CAI (Shmueli-Goetz et al., 2004, unpublished) is a semi-structured interview assessing children’s attachment representations of their current relationships with their primary caregivers. The interview is similar in format, scope, and theoretical underpinnings to that employed in the Adult Attachment Interview (AAI; George et al., 1985, unpublished), but all of these elements of assessing attachment representations are developmentally scaled for measurement in middle childhood (ages 7–12). Children are asked to describe their relationships with their primary caregivers and to support their descriptions by providing examples of concrete relationship episodes. Trained CAI coders rate the CAI using both verbatim transcripts of CAI narratives and videotapes using 10 nine-point scales capturing different aspects of security (e.g., emotional openness, preoccupied anger, idealization, and narrative coherence). Based on these scales, coders assign children to one of four attachment categories with respect to each parent: secure, dismissing, preoccupied, and disorganized. In prior studies, most children with two caregivers receive the same classification for both (e.g., Shmueli-Goetz et al., 2008).

The CAI demonstrates concurrent validity in community, clinical, and CSA samples (Target et al., 2003; Shmueli-Goetz et al., 2008; Scott et al., 2011; Venta et al., 2014; Borelli et al., 2016a,b; Ensink et al., 2016a,b), and inter-rater reliability among expert and “naive” coders is acceptable (e.g., Shmueli-Goetz et al., 2008; see Privizzini, 2017, for a review).

In the current study, the interviewers and coders were trained by an expert coder (first author) to reliability, who also supervised their work. All the transcripts were double coded, and where there was coder disagreement, the first author recoded the transcript and a consensus decision was reached after clarifying and examining reasons for lack of agreement (four-way classification: *k* = 0.91, *p* = 0.001).

#### Trauma-Related Psychopathology

Children reported on their psychopathology symptoms using the Trauma Symptoms Checklist for Children (TSCC; Briere, 1996), a 54-item checklist administered in an interview format. The TSCC was designed to measure the severity of posttraumatic stress disorder (PTSD) and associated psychological symptomatology in children aged 8–16 years and is the most widely used, standardized and normed measure of trauma related symptomatology in children (Elhai et al., 2005; Lanktree et al., 2012). The TSCC is comprised of six scales covering anxiety, depression, anger, post-traumatic stress, dissociation, as well as sexual concerns. For each item, the child indicates the frequency with which the statement pertains to her/him on a four-point scale ranging from 0 (never) to 3 (almost all the time). Raw scale scores are derived by summing the response values for all items comprising the scale, and then dividing by the number of items in the scale. A higher score reflects greater symptomatology. Alpha coefficients for clinical scales ranged from 0.77 to 0.89. Raw scores are converted to standardized *t*-scores.

### Data Analytic Plan

In all analyses, we used *t*-scores of child-reported symptoms, which are scores that are adjusted for children’s sex and age norms, thereby eliminating the need to control for children’s age and sex in analyses. To test study hypotheses, we used hierarchical linear regressions involving tests of interaction effects – we employed Hayes’ PROCESS Macro (Model 1), which uses 5,000 bootstrapping samples to estimate the association between *x* and *y* low (−1 SD below the mean), mean, and high (+1 SD above the mean) levels of *m*.

In all analyses, we included as independent variables CSA exposure (CSA vs. no-CSA) and attachment classification (secure vs. insecure), as well as the interaction between abuse status and attachment classification, in the prediction of trauma-related psychopathology.

To determine the distribution of the study variables, we examined the descriptive statistics of the sample in children with intra-familial CSA as well as extra-familial CSA. Specifically, we conducted independent samples *t*-tests and found that the levels of child reported psychopathology across intra-familial and extra-familial CSA did not significantly differ on any scale,^1^ allowing us to collapse CSA scores into one overall CSA group.

Next, we examined the associations between demographic and key study variables through conducting a series of bivariate correlations. Associations between CSA and attachment were determined through acknowledging attachment as a two-level variable – secure vs. insecure. Trauma related psychopathology scores were consolidated based on the six clinical subscales of the TSCC (Table 2).

**Table 2:** Bivariate correlations among demographic variables and key study variables.

S. No.		2	3	4	5	6	7	8	9	10	11	12	13
1.	Child age	−0.07	−0.05	0.11	−0.24^*^	0.00	−0.21^*^	−0.15	−0.14	−0.10	0.05	−0.07	0.10
2.	Child sex	---	0.05	0.06	−0.04	−0.06	0.03	−0.14	−0.16	−0.08	0.03	−0.13	−0.11
3.	Mother educational attainment		---	0.39^**^	−0.22^*^	−0.29^**^	−0.21^*^	−0.13	−0.10	−0.14	−0.10	−0.11	−0.17
4.	Household income			---	−0.43^**^	−0.23^*^	−0.26^**^	−0.12	−0.11	−0.12	−0.19	−0.24^*^	−0.12
5.	Children’s stress exposure				---	0.19^*^	0.26^**^	0.05	0.08	−0.03	0.01	−0.03	0.10
6.	Children’s CSA exposure					---	0.36^**^	0.00	0.07	0.17	0.15	0.05	0.18
7.	Children’s attachment classification						---	0.14	0.13	0.197^*^	0.23^*^	0.12	0.17
8.	Posttraumatic symptoms							---	0.67^**^	0.70^**^	0.49^**^	0.71^**^	0.55^**^
9.	Anxiety symptoms								---	0.66^**^	0.53^**^	0.58^**^	0.61^**^
10.	Depression symptoms									---	0.69^**^	0.61^**^	0.61^**^
11.	Anger symptoms										---	0.44^**^	0.51^**^
12.	Overt dissociation symptoms											---	0.45^**^
13.	Sexual concern symptoms												---

* *p* < 0.05;

** *p* < 0.01.

To test study hypotheses, we conducted a series of hierarchical linear regressions in which we explored whether the association between children’s CSA exposure and children’s trauma-related psychopathology varied as a function of children’s attachment security. To test interaction effects, we used Hayes’ PROCESS (2012) Macro for SPSS, Model 1, which tabulates the slopes of *x* on y at −1, mean, and +1 SD values of the moderator using bootstrapping.

## Results

Bivariate correlations revealed that mothers of children with CSA, as well as mothers of children with insecure attachment reported lower household incomes. Children whose families reported lower levels of household income and lower maternal education, as well as children who were younger, had higher levels of exposure to stressors. Younger children were more likely to be classified as having insecure attachment and children from families with lower household incomes had more overt symptoms of dissociation. Unsurprisingly, children in the CSA-exposed group had higher levels of overall stress exposure, underscoring the need to control for stress exposure in hypothesis testing. In addition, we also controlled for household income and maternal education, given the association between these variables and key constructs of interest. Interestingly, the bivariate correlations did not reveal significant associations between either attachment classification or CSA history with child reports of psychopathology.

### Testing the Unique and Interactive Associations Between CSA and Insecure Attachment in Predicting Trauma-Related Pathology

#### PTSD Symptoms

**Figure 1:**
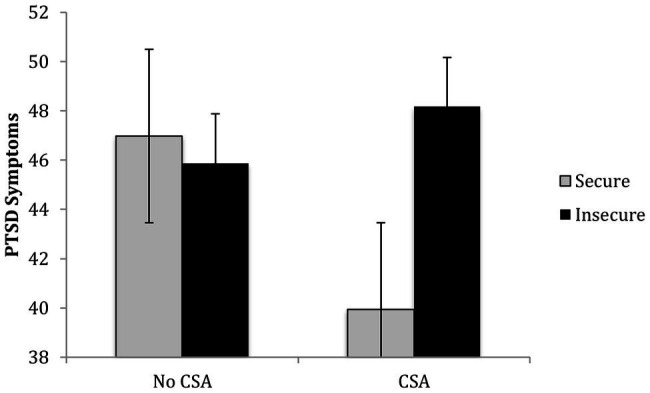
Children’s childhood sexual abuse (CSA) history and attachment interact in predicting their post-traumatic stress disorder (PTSD) symptoms.

The results of regression analysis revealed that after controlling for covariates and the main effects of CSA and attachment, *R*^2^ = 0.09, *p* = 0.14, the second step containing the CSA x attachment interaction significantly contributed to the prediction of children’s PTSD symptoms, Δ*R^2^* = 0.05, *p* = 0.02. Examination of the simple slopes revealed that CSA-exposed children showed a significant association between insecure attachment and greater PTSD symptoms, *b* = 8.23, *p* = 0.01, whereas among comparison group children, attachment was not significantly associated with PTSD symptoms, *b* = −1.09, *p* = 0.63 (see Figure 1).

#### Anxiety Symptoms

**Figure 2:**
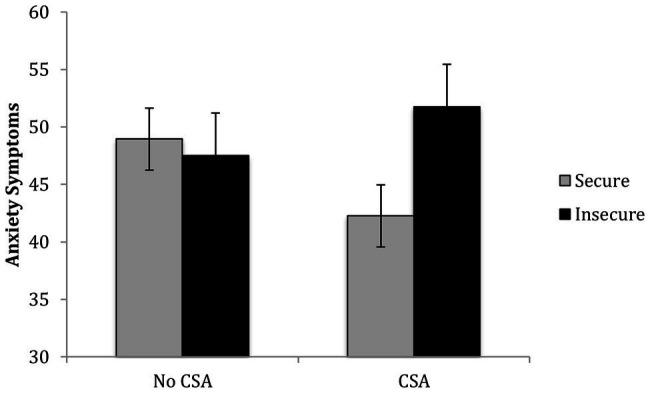
Children’s CSA history and attachment interact in predicting their anxiety symptoms.

The results of a regression revealed that after controlling for covariates and the main effects of CSA and attachment, *R*^2^ = 0.08, *p* = 0.23, the second step containing the CSA x attachment interaction significantly contributed to the prediction of children’s anxiety symptoms, Δ*R*^2^ = 0.05, *p* = 0.02. Among abused children, insecure attachment was associated with higher anxiety symptoms, *b* = 9.49, *p* = 0.01, whereas among comparison group children, attachment was not associated with anxiety, *b* = −1.43, *p* = 0.60 (see Figure 2).

#### Depressive Symptoms

**Figure 3:**
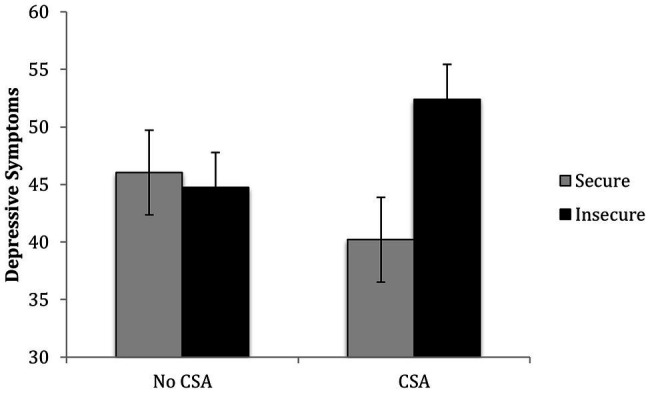
Children’s CSA history and attachment interact in predicting their depression symptoms.

The results of a regression revealed that after controlling for covariates and the main effects of CSA and attachment, *R*^2^ = 0.14, *p* = 0.02, the second step containing the CSA x attachment interaction significantly contributed to the prediction of children’s depressive symptoms, Δ*R*^2^ = 0.07, *p* = 0.01. Among CSA-exposed children, insecure attachment was associated with higher depressive symptoms, *b* = 12.19, *p* = 0.002, whereas among comparison group children, attachment was not associated with depression, *b* = −1.31, *p* = 0.65 (see Figure 3).

#### Anger Symptoms

**Figure 4:**
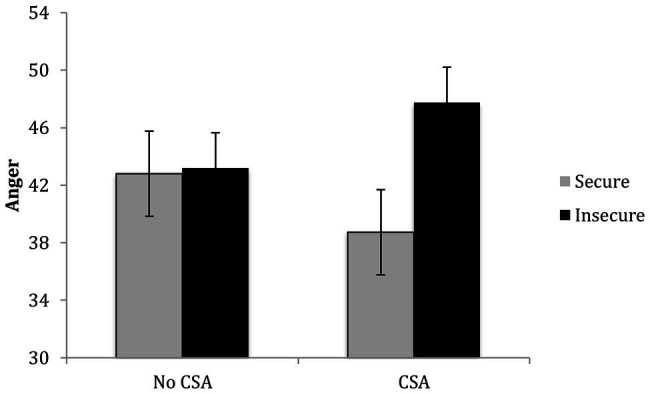
Children’s CSA history and attachment interact in predicting their anger symptoms.

The results of a regression revealed that after controlling for covariates and the main effects of CSA and attachment, *R*^2^ = 0.14, *p* = 0.02, the second step containing the CSA x attachment interaction significantly contributed to the prediction of children’s anger symptoms, Δ*R*^2^ = 0.04, *p* = 0.03. Among CSA-exposed children, insecure attachment was associated with greater anger, *b* = 9.03, *p* = 0.004, but among comparison group children, insecure attachment was not associated with greater anger, *b* = 0.40, *p* = 0.86 (see Figure 4).

#### Dissociation

**Figure 5:**
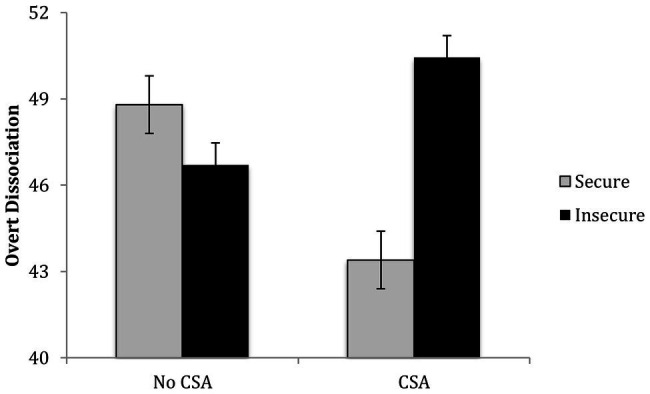
Children’s CSA history and attachment interact in predicting their overt dissociation.

The results of a regression revealed that after controlling for covariates and the main effects of CSA and attachment, *R*^2^ = 0.13, *p* = 0.03, the second step containing the CSA x attachment interaction significantly contributed to the prediction of children’s overt dissociation symptoms, Δ*R*^2^ = 0.05, *p* = 0.02. Among abused children, insecure attachment was associated with more dissociation, *b* = 7.05, *p* = 0.02, but among comparison children, attachment was not significantly associated with dissociation, *b* = −2.08, *p* = 0.37 (see Figure 5).

#### Sexual Concerns

The results of a regression revealed that after controlling for covariates and the main effects of CSA and attachment, *R*^2^ = 0.05, *p* = 0.46, the second step containing the CSA × attachment interaction did not significantly contribute to the prediction of children’s sexual distress symptoms, Δ*R*^2^ = 0.02, *p* = 0.19.

### Multivariate Follow-Up Analysis

To account for the positive intercorrelations among the dependent variables (children’s self-reported psychopathology symptoms), we followed up the individual analyses by conducting a multivariate analysis in which we included all six clinical subscales from the TSCC as dependent variables. This multivariate analysis of covariance uses a general linear model framework and enables the inclusion of multiple categorical and continuous criterion variables in the prediction of multiple dependent variables. In this analysis, we controlled for the same three covariates and then included the main effects and the interaction term in the model. The results were almost identical to those obtained from the models involving the individual clinical scales, suggesting that the interaction of CSA and attachment significantly predicted children’s PTSD, *p* = 0.02, anxiety, *p* = 0.02, depression, *p* = 0.007, anger, *p* = 0.03, and dissociation, *p* = 0.02, but not sexual distress, *p* = 0.20. That the effects emerging from this multivariate model so closely paralleled the other findings enhanced our confidence in the original findings.

## Discussion

The aim of the present study was to investigate the interactions between CSA and attachment security as risk and protective factors for PTSD and other trauma related symptoms in school aged children. The findings indicate that CSA and attachment interacted so that child CSA survivors with secure attachment representations had significantly lower PTSD symptoms, while CSA survivors with insecure attachment representations were at elevated risk. This pattern was also evident for other trauma related symptoms; CSA survivors with insecure attachment representations manifested significantly more depressive symptoms, anxiety symptoms, dissociative symptoms, somatic symptoms and reported more trauma related anger. The pattern of findings was particularly striking, especially because neither CSA history nor attachment insecurity themselves were uniquely associated with child self-reported psychopathology on the Trauma Symptom Inventory. Instead, it was only in interaction with one another that the associations emerged. This pattern of effects, which was contrary to our predictions, is interesting in light of the substantial body of evidence linking both CSA and attachment insecurity independently to psychopathology within this age range (e.g., Briere et al., 2008), whereas in the current study, neither risk factor alone was sufficient to drive an association between the two. The association between attachment and trauma symptoms was uniquely observed in the group of child CSA survivors indicating that it is in the context of trauma that attachment security is associated with how children process trauma. In the non-abused comparison group attachment was not associated with PTSD and trauma symptoms. In sum, the findings indicate that attachment moderates the relation between CSA and child reports of PTSD as well as other trauma related symptoms. This draws attention to the importance of secure child attachment relationships and IWMs for children’s recovery after trauma. From a theoretical perspective the findings that CSA survivors with insecure attachment were at heightened risk of PTSD and other trauma symptoms, are consistent with propositions that secure attachment may have restorative effects after trauma and could be seen as a protective shield (Lieberman and Amaya-Jackson, 2005). It also adds to the growing body of findings that attachment influence how traumatic reactions are experienced and expressed (Mikulincer et al., 2015) and impacts the development of PTSD in adults (Woodhouse et al., 2015; Barazzone et al., 2019). In sum, it provides further evidence of the importance of “using a dual lens” on trauma and attachment (Lieberman and Amaya-Jackson, 2005) and to use an approach that considers both of these important influences on child development.

The findings that child attachment moderates the relation between CSA and child reports of PTSD and trauma symptoms, provides new evidence of the importance of attachment for school aged children exposed to trauma. The findings of the present study suggest that the protective effects of secure attachment in the context of trauma that has previously been observed in adults and adolescents are also present for children. It is in line with previous findings with adolescents showing that attachment moderates the relation between CSA and trauma symptoms (Jardin et al., 2017), as well as findings with adults that attachment security is a protective factor against the development of trauma symptoms (Aspelmeier et al., 2007), and that attachment mediated the relationship between CSA and trauma symptoms (Roche et al., 1999). The findings that child CSA survivors with secure attachment have a lower risk of manifesting PTSD and other trauma related symptoms, while child CSA survivors with insecure attachment appear particularly at risk, adds to our knowledge and helps to elucidate the processes that mediate and moderate the associations between CSA and a range of child psychological difficulties. It extends previous findings with school aged children showing that insecure attachment is a risk factor for child reported depressive symptoms in both child CSA survivors as well as in non-abused children (Ensink et al., 2020). In that study, school aged CSA survivors were found to be more likely to have insecure and disorganized attachments to parents than non-abused children, and that insecure attachment was the only factor associated with higher self-reported depressive symptoms in both child survivors of CSA and the comparison group. In addition, the findings of the present study showing that secure attachment is associated with fewer PTSD symptoms as well as other trauma symptoms also extend the findings of a number of studies showing that children’s mentalizing regarding themselves and attachment figures, an ability closely related to attachment, mediates the relationship between CSA and child outcome such as child depressive symptoms as well as externalizing symptoms (Ensink et al., 2016a, 2017).

We followed up our initial regressions by conducting a MANCOVA, which enabled us to account for the fact that we were conducting multiple analyses by controlling for all dependent variables within the same analysis. Our findings within this MANCOVA revealed that all effects remained significant with the exception of the sexual concerns effect. This leads us to conclude that the interaction between CSA and insecure attachment with sexual problems does not hold after controlling for all of the other clinical scales, suggesting that it may be weaker. We reason that this may be due to the developmental stage of the children examined in this investigation, and we wonder what pattern of effects would emerge were the study conducted with adolescents who had experienced CSA.

Given the clinical implications of the findings, it is important to consider likely processes through which attachment impact manifestations of PTSD and trauma symptoms. There are a number of possible explanations for why child CSA survivors with secure attachments report fewer PTSD symptoms as well as other trauma related symptoms. Models of pathways involving for example attachment and emotion regulation strategies have been tested in adults (O’Connor and Elklit, 2008). Similar research with children is lagging and adult models do not necessarily apply to children given that they still partially depend on parents for a sense of safety, support, understanding and help with regulating emotions when trauma overwhelms their own developing capacities for emotion regulation. Based on existing evidence, three key interconnected processes may account for why child CSA survivors with secure attachments to parents manifest fewer PTSD and trauma symptoms. Being able to access secure attachment relationships and turn to parents for safety when trauma activates children’s need for safety, is likely particularly important for child recovery after trauma. Support from parents and strong peer relationships, have been shown to have important buffering effects subsequent to sexual abuse. Parental reflective functioning about the child, is known to be associated with secure child attachment, and has been shown to be associated with more positive interactions with children, and a better ability to understand and imagine what is in the child’s mind and is associated with better child psychological functioning subsequent to sexual abuse (Ensink et al., 2017).

Furthermore, attachment models of the processes through which attachment impacts the health and mental health of adults put forth that secure attachment decreases vulnerability to stress because it underlies more efficient emotion regulation strategies (O’Connor and Elklit, 2008). This pathway still needs to be directly tested in children exposed to trauma. However, there is evidence that insecure attachment is associated with non-optimal emotional regulation strategies involving upregulation or avoidance, while secure attachment is associated with an improved ability to downregulate and/or reappraise stress responses. The latter has been found to act as a protective factor against fear and physiological dysregulation (Parrigon et al., 2015) and negative reactions to life events (Feeney, 2000; Maunder and Hunter, 2001; Pietromonaco et al., 2013). Better emotion regulation and associated brain functioning has been linked to resilient functioning after childhood trauma (Daniels et al., 2012). Mentalization models of attachment is also closely associated with mentalization and social-cognition. The importance of mentalizing for child recovery after trauma has been demonstrated in a number of studies (Ensink et al., 2016a, 2017). In addition, our previous studies with school aged children with CSA indicate that mentalizing, an ability overlapping with attachment and referring to children’s capacity to think of themselves and others in mental state terms, mediate the relationship between CSA and outcomes such as externalizing and depressive symptoms. Children’s mentalizing has also been shown to predict cardiovascular regulation following stress (Borelli et al., 2018). Our understanding of this is that child attachment is associated with emotion regulation, underlies children’s representations of self and others around which their sense of self and identity is constituted and which underlies their capacity to understand their own reactions and that of others in terms of underlying mental states. Mentalizing and the capacity to identify and verbally express affects and think about the intentions of others may be particularly important in processing and regulating fear and anger.

The present study has a number of strengths, including the use of interview measures with children to evaluate child attachment as well as child reports of PTSD and trauma related symptoms. Furthermore, the study focuses on a difficult-to-recruit but important clinical population. Further strengths include the use of a comparison group, as well as the inclusion of children with intrafamilial CSA and extrafamilial CSA. Although the sample was comparable in size to that of other studies of attachment in school-age children, it remains small to detect effects of this magnitude (Δ*R*^2^ values between 0.02 and 0.07), indicative of small to medium effect sizes. In addition, CSA-exposed children are also more likely to experience other forms of psycho-social adversity; to address this potential confound in the associations examined in this study, we controlled for children’s stress exposure, but we acknowledge that this cannot completely address the potential influence of co-occurring risk factors on children’s adjustment. Further, the children in this study experienced a range of types and levels of CSA seen in a community sample, consistent with the definition of sexual abuse of Putnam (2003). This may be different from the experiences of CSA survivors put into the care of child protective services, who may have experienced severe, chronic CSA together with neglect, physical and psychological abuse. Given that the children in our sample were referred by health services rather than child protective services, it was not possible to control for possible co-occurring abuse and neglect, as this information could not be reliably collected from parents or children. We were not able to examine the role of children’s disclosure of CSA or parents’ reactions to children’s disclosure on outcomes, which is also an area in need of additional investigation. Further research is needed to examine attachment in different samples of children with CSA. Finally, the cross-sectional nature of the study limits the extent to which a temporal developmental sequence can be inferred.

## Conclusion

In sum, the findings reveal that a “dual lens” on trauma and attachment can assist in identifying protective and aggravating processes linking these two major influences on children’s developmental course. Attachment moderated the relationship between CSA and child reported PTSD and trauma related symptoms; child CSA survivors with insecure attachment to their parents reported significantly more PTSD symptoms, depression, anxiety, dissociation, and anger. Child CSA survivors with insecure attachments are at particular risk of manifesting PTSD and trauma related symptoms in contrast with child survivors with secure attachment to parents. These findings showing that child CSA survivors with insecure attachment are at elevated risk have important implications for identifying child CSA survivors most in need of treatment. As importantly, the findings suggest that attachment needs to be incorporated in our clinical formulations and treatment plans of child CSA survivors.

## Data Availability Statement

The original contributions presented in the study are included in the article/supplementary material, further inquiries can be directed to the corresponding author.

## Ethics Statement

The studies involving human participants were reviewed and approved by University Laval Ethics Committee. Written informed consent to participate in this study was provided by the participants’ legal guardian/next of kin.

## Author Contributions

KE: theoretical framework, writing, conception, execution of study, and interpretation of results. JB: conception and execution of data analyses and interpretation of results. LN: principal investigator. AR: literature review and data analysis. CM: literature review and data analysis. PF: theoretical contribution. All authors contributed to the article and approved the submitted version.

## Conflict of Interest

The authors declare that the research was conducted in the absence of any commercial or financial relationships that could be construed as a potential conflict of interest.

## Publisher’s Note

All claims expressed in this article are solely those of the authors and do not necessarily represent those of their affiliated organizations, or those of the publisher, the editors and the reviewers. Any product that may be evaluated in this article, or claim that may be made by its manufacturer, is not guaranteed or endorsed by the publisher.

## Footnotes

^1^PTSD: *t*(40) = 0.72, *p* = 0.40; anxiety: *t*(40) = âˆ’0.66, *p* = 0.51; depressive: *t*(40) = 0.13, *p* = 0.99; dissociation: *t*(40) = 0.68, *p* = 0.49; and sexual concerns: *t*(40) = 0.51, *p* = 0.61.
